# The role of the different CD3γ domains in TCR expression and signaling

**DOI:** 10.3389/fimmu.2022.978658

**Published:** 2022-09-02

**Authors:** Beatriz Garcillán, Rebeca F. Megino, Marta Herrero-Alonso, Alberto C. Guardo, Veronica Perez-Flores, Claudia Juraske, Vincent Idstein, Jose M. Martin-Fernandez, Carsten Geisler, Wolfgang W. A. Schamel, Ana V. Marin, Jose R. Regueiro

**Affiliations:** ^1^ Department of Immunology, Ophthalmology and Ear, Nose and Throat (ENT), Complutense University School of Medicine and 12 de Octubre Health Research Institute (imas12), Madrid, Spain; ^2^ Signaling Research Centers BIOSS and CIBSS, University of Freiburg, Freiburg, Germany; ^3^ Department of Immunology, Faculty of Biology, University of Freiburg, Freiburg, Germany; ^4^ Centre for Chronic Immunodeficiency (CCI), Medical Center Freiburg and Faculty of Medicine, University of Freiburg, Freiburg, Germany; ^5^ Spemann Graduate School of Biology and Medicine (SGBM), University of Freiburg, Freiburg, Germany; ^6^ The LEO Foundation Skin Immunology Research Center, Department of Immunology and Microbiology, Faculty of Health and Medical Sciences, University of Copenhagen, Copenhagen, Denmark

**Keywords:** CD3 chimeras, T cell receptor, CD3δ, CD3γ, domains

## Abstract

The CD3 subunits of the T-cell antigen receptor (TCR) play a central role in regulation of surface TCR expression levels. Humans who lack CD3γ (γ^—^) show reduced surface TCR expression levels and abolished phorbol ester (PMA)-induced TCR down-regulation. The response to PMA is mediated by a double leucine motif in the intracellular (IC) domain of CD3γ. However, the molecular cause of the reduced TCR surface expression in γ^—^ lymphocytes is still not known. We used retroviral vectors carrying wild type CD3γ or CD3δ or the following chimeras (EC-extracellular, TM-transmembrane and IC): δ_EC_γ_TM_γ_IC_ (δγγ for short), γγδ, γδδ and γγ-. Expression of γγγ, γγδ, γδδ or γγ- in the γ^—^ T cell line JGN, which lacks surface TCR, demonstrated that cell surface TCR levels in JGN were dependent on the EC domain of CD3γ and could not be replaced by the one of CD3δ. In JGN and primary γ^—^ patient T cells, the tested chimeras confirmed that the response to PMA maps to the IC domain of CD3γ. Since protein homology explains these results better than domain structure, we conclude that CD3γ contributes conformational cues that improve surface TCR expression, likely at the assembly or membrane transport steps. In JGN cells all chimeric TCRs were signalling competent. However, an IC domain at CD3γ was required for TCR-induced IL-2 and TNF-α production and CD69 expression, indicating that a TCR without a CD3γ IC domain has altered signalling capabilities.

## Introduction

The αβ T cell receptor (TCR) consists of the disulfide linked variable TCRαβ heterodimer and the non-covalently associated CD3γ, CD3δ, CD3ε and ζ invariant chains (1, 2). The function of the TCRαβ dimer is to recognize antigens on the surface of antigen presenting cells, whereas the role of invariant chains is to mediate activation signals and to regulate the level of TCR expression (3, 4). TCR chains are type I integral membrane proteins, and, except for ζ, belong to the immunoglobulin superfamily. All TCR chains were initially believed to be necessary for efficient TCR expression at the cell surface (5) but the study of TCR immunodeficiencies (TCRID) has demonstrated that this is not the case, as some TCRID show a significant number of T cells able to express a surface TCR despite the lack of certain TCR subunits, such as CD3γ (6–8).

For example, CD3γ and CD3δ play a role in the regulation of TCR expression levels on the cell surface. They arose from a gene duplication event in mammals and are thus highly homologous, especially in the intracellular (IC) domain. However, their specific contribution to regulate TCR expression is still unclear. Humans lacking CD3γ (γ^—^) show reduced surface TCR expression levels associated to a potentially lethal immunodeficiency and/or autoimmunity of variable severity (9, 10). In these T cells in which the CD3γ is substituted by another CD3δ (11), phorbol ester acetate (PMA)-induced TCR internalization is not taking place, but antigen-induced TCR internalization does (12). Indeed, TCR downmodulation in response to PMA has been mapped to the IC domain of CD3γ. The IC domain of CD3γ has been also implicated in TCR trafficking, as TCR triggering induces down-modulation to regulate T-cell responses, phosphorylation of a serine residue in the cytoplasmic tail of CD3γ was required for this process, and a di-leucine motif in the cytoplasmic tail of CD3γ was required for PKC-mediated TCR downregulation (13). However, the molecular cause of the reduced expression of the TCR on the surface of unstimulated γ^—^ T cells is still unknown.

The lack of CD3ε or ζ leads to the absence of TCR cell surface expression (14–16). CD3δ and CD3γ, on the other hand, can be dispensable for TCR cell surface expression, since transfected mouse cells expressing all TCR components, except CD3δ or CD3γ, and T cells of CD3γ-deficient patients still express substantial surface TCR (16, 17). Since other cases have been described where surface TCR expression does depend on CD3δ and CD3γ (18, 19), the rules governing TCR export are apparently somewhat variable with respect to these two components.

Thus, although the structure of the extracellular (EC) and transmembrane (TM) regions of the TCR were solved recently (20), it is still unclear how CD3δ and CD3γ can substitute for each other in the assembly of the TCR. Studies in a T-cell line called JGN (Jurkat Gamma Negative) (19), demonstrated that the EC domain of CD3δ cannot substitute for the EC domain of CD3γ in TCR expression (21). This indicates that the EC domain of CD3γ has a specific role in TCR assembly in JGN. The TM domain is also essential for the formation of the eight-chain TCRαβ-CD3εδ-CD3εγ-ζζ complex, and assembly is dependent on proper placement of three ionizable TM residues (20, 22).

The aim of this study was to analyze the importance of each domain of CD3γ in a clonal cell line lacking this chain, JGN, or in natural and polyclonal patient-derived γ^—^ cells. We have done this in comparison with the highly homologous domains of CD3δ to avoid side effects derived from steric discrepancies or any other features that could affect our conclusions about the uniqueness of CD3γ. The two cell models (JGN and γ^—^ T cells) have been used to analyze the role of CD3γ in human T lymphocytes, but no side-by-side comparison had been done to date to uncover any possible disparities.

## Materials and methods

### Cell isolation and culture

Blood samples were obtained from a γ^—^ patient (FK, family 2, IV.4, homozygous for c.205A>T/p.K69X) (7) or from healthy volunteers. Peripheral blood lymphocytes (PBL) were collected after gradient centrifugation in Ficoll-Paque™ (GE Healthcare).

JGN cells, a TCR cell surface negative variant of the human T-cell line Jurkat that lacks CD3γ (19) and the original CD3γ+TCR+ Jurkat cell line J76, were maintained in RPMI 1640 medium (HyClone, Logan, UT, United States) containing 10% FCS, 1% antibiotic/antimycotic and 1% L-glutamine (both from Life Technologies, Carlsbad, CA, United States).

PG13 cells were grown in DMEM (Gibco, Waltham, MA, United States) supplemented with 10% FCS, 1% L-glutamine and 1% antibiotic/antimycotic. Transduced cells were grown in antibiotic-containing selection media containing 1 μg/mL puromycin from Sigma-Aldrich (St. Louis, MO, United States).

### Retroviral vectors and transduction

cDNA constructs carrying distinct domains (EC, TM or IC) of CD3γ or CD3δ, the wild type chains or p.M1V (named as M1V) and p.H29X (as H29X) protein-null mutations in *CD3G* (9, 23, 24) were generated and cloned in the LZRS-EGFP bicistronic retroviral vector as previously described (21, 25). PG13 packaging cells were transfected with these vectors and selected for puromycin resistance and EGFP expression. Vector-containing retroviral supernatants were harvested, filtered and used immediately.

For retroviral transduction, PBL or JGN cells were plated on 24-well plates precoated with the CH296 recombinant fibronectin fragment from TaKaRa Bio (Japan) at 2 x 10^6^ cells/mL in supplemented RPMI 1640 medium containing 80 IU/mL rhIL-2 and retroviral supernatants at 1:1 proportion. Prior to transduction, PBL were activated for 48 hours (h) with 80 IU/mL recombinant human IL-2 and 1 μg/mL Phytohemagglutinin-L (Sigma-Aldrich); for JGN cells activation was not necessary. After 16 h of exposure to retrovirus, the cells were centrifuged and resuspended in fresh medium. After 72 h, EGFP expression was determined by flow cytometry to monitor transduction efficiency.

### Flow cytometry

Cells were stained with monoclonal antibodies (mAb) against CD3 (SK7 and Leu-4 clones) and CD69 (L-78) from Becton Dickinson (Franklin Lakes, NJ, United States), CD3 (UCHT-1) from Beckman Coulter (Brea, CA, United States), CD3 (S4.1) from Life technologies (Carlsbad, CA, United States). For unlabeled antibodies, a second step with PE-conjugated anti-mouse secondary antibody (Beckman Coulter) was performed. Extracellular staining was performed following standard procedures. For intracellular staining, cells were fixed with p-formaldehyde 4% and then permeabilized with 0.5% saponin (Sigma-Aldrich). Gating was always done in the lymphocyte region. Data were acquired with a FACSCalibur flow cytometer (Becton Dickinson) and analyzed with FlowJo software (TreeStar, Ashland, OR, United States).

### TCR internalization assays

Phorbol 12-myristate 13-acetate (PMA)-induced TCR internalization was measured after stimulation with 20 ng/mL PMA (Sigma Aldrich) for 30 minutes by surface flow cytometry using anti-CD3 mAb (SK7).

For CD3-mediated TCR internalization, cells were plated at 10^6^ cells/mL in 96-well plates and incubated for 24 h in the absence (negative control) or presence of immobilized anti-CD3 mAb (Leu4), and surface TCR levels were analyzed by surface flow cytometry using anti-CD3 mAb (SK7).

### TCR functional assays

For CD69 upregulation, cells were plated at 10^6^ cells/mL in 96-well plates and incubated for 24 h in the absence (negative control) or presence of immobilized anti-CD3 mAb (Leu4).

Cytokine production was measured using human-Intracellular Cytokine Staining Starter Kit from Becton Dickinson following the manufacturer´s instructions. Briefly, resting cells were resuspended at 10^6^ cells/mL, seeded in 96-well plates, and stimulated for 6 h with immobilized anti-CD3 mAb (IOT3b, 1 μg/mL; Beckman Coulter). For the last 2 h of stimulation, 10 μg/mL brefeldin A (Sigma Aldrich) were added to the cultures. Stimulated cells were harvested, washed twice in phosphate buffered saline (PBS), fixed, permeabilized and incubated with anti-IL-2 or anti-TNF-α PE mAb. The cytometric analyses were performed in a FACSCalibur BD analyzer as described above.

For CD3ε tyrosine phosphorylation after anti-CD3 stimulation, cells were stimulated at 6x10^6^ cell/mL with 5 μg UCHT-1 at 37°C for 2 to 30 minutes, then washed in cold PBS and lysed with 500 μL cold lysis solution (1% NP-40, 140 mM NaCl, 10 mM Tris pH 7.5, 5 mM EDTA, 10 mM PMSF, 1 μg/mL aprotinin and 1 mM sodium orthovanadate) for 30 minutes. Lysates were centrifuged at 12,000 g, 30 minutes and supernatants were subjected to SDS-PAGE in 7.5% gels. Proteins were transferred to a PVDF membrane from Bio-Rad (Hercules, CA, United States) for 30 minutes in a semi-dry transfer system at 18 V (Bio-Rad). Membranes were revealed with rabbit anti-phospho-CD3ε-Y1 antisera, kindly provided by Wolfang Schamel, Department of Immunology, Faculty of Biology, University of Freiburg, Germany, and rabbit anti-ζ antisera (448), kindly provided by Balbino Alarcón, Centro de Biología Molecular Severo Ochoa, UAM-CSIC, Madrid, Spain. Further analysis was done in an Odyssey Infrared Imaging System (LI-COR Biosciences, Lincoln, NE, United States).

### TCR structural assays

The CD3 Conformational Change (CC) was measured by Nck pull-down (PD). Briefly, JGN cells were stimulated with 10 μg/mL anti-CD3 mAb (UCHT-1) or anti-TCR Vβ8 (56C5.2) for 5 minutes at 37°C and lysed in 1 mL lysis buffer (0.5% Brij96V from Sigma-Aldrich, 20 mM Tris–HCl pH 8, 150 mM NaCl, 10% glycerol, 4 mM EDTA, 1 mM PMSF, 2 mM Na_3_VO_4_, 10 mM NaF, and 1x Protease Inhibitor Cocktail Set I—Calbiochem). Glutathione-S-transferase (GST) sepharose beads bound to the SH3.1 domain of Nck (GST-Nck^SH3.1^) have been previously described (26). Cell lysates were incubated with the beads for 3 h at 4°C. After the PD, beads were analyzed by flow cytometry or western blot to detect the attached TCRs that had undergone the CC. For flow cytometry (27), beads were probed with anti-TCR Vβ8 PE (Beckman Coulter). For western blot analysis half of the lysate was used for the PD and the other half was used to confirm antibody binding of the stimulating antibodies by immunoprecipitations using protein G-coupled Sepharose beads (Amersham) as described (28).

To evaluate TCR of each cell line to organize in nanoclusters, cells were lysed in 1 mL of lysis buffer containing 20 mM TrisHCl, pH 8, 137 mM NaCl, 2 mM EDTA, 10% glycerol, 10 μg/mL leupeptin, 10 μg/mL aprotinin, 1 mM PMSF, 500 μM sodium orthovanadate, 1 mM NaF, and the appropriate detergent (1% digitonin to reveal monomeric TCR or 0.5% Brij96V to monomeric and nanoclustered TCRs). Lysates were separated by reducing SDS-PAGE and proteins were transferred to PVDF or nitrocellulose membranes under standard conditions. Monomeric and nanoclustered TCRs were analyzed by Blue Native PolyAcrylamide Gel Electrophoresis (BN-PAGE) and anti-ζ immunoblotting, preparation of membrane fractions and BN-PAGE were performed as described (28). Immunoblotting was performed according to standard protocols using anti-CD3ε antisera (M20) from Santa Cruz, anti-BAP32/37 (29) and anti-ζ antisera (448) were previously described (30).

### Statistical analysis

Student’s t-test was performed using SPSS statistical software. Only p values below 0.05 were considered significant. Data are presented as mean ± SEM (standard error of the mean) or ± SD (standard deviation).

## Results

### The EC domain of CD3γ is required for TCR cell surface expression in JGN cells but not in T cells from γ^—^ patients

**Figure 1:**
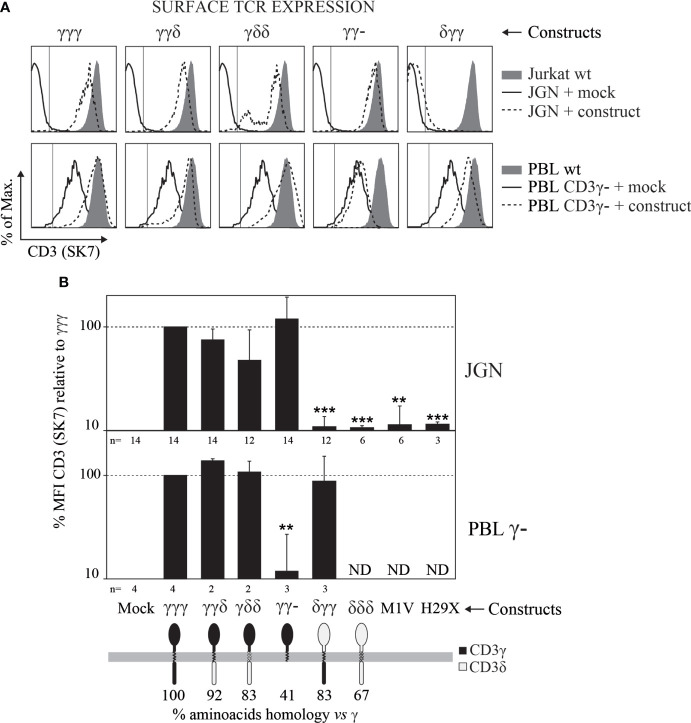
Surface TCR expression determined by flow cytometry in γ^—^ cells (JGN or PBL) transduced with the indicated CD3γ/δ constructs. **(A)** Representative CD3 expression histograms in cells transduced with the indicated constructs (dashed lines) in comparison with Mock-transduced cells (solid lines) and their respective normal (γ^+^) controls, Jurkat J76 for JGN cells and normal PBL for natural γ^—^ cells (filled histograms). **(B)** CD3 mean fluorescence intensity (MFI) relative to that of γγγ ± SEM in three independent experiments. ** *p*<0.01, *** *p*<0.001. n indicates number of repetitions with each cell line. Additional anti-TCR antibodies were studied in JGN constructs transduced cells obtaining similar results (see **Supplementary Figure S1**).

To study the contribution of the different domains of CD3γ in TCR cell surface expression, we used the γ^—^ mutant Jurkat T-cell line JGN and γ^—^ T cells from CD3γ-deficient patients. These T cells were retrovirally transduced with plasmids encoding for CD3γ/δ chimeric constructs that carry distinct domains: δ_EC_γ_TM_γ_IC_ (δγγ for short), γγδ and γδδ. Wild type (γγγ), IC-truncated (γγ-) or protein-null mutants (M1V, H29X) CD3γ, as well as wild type CD3δ (δδδ) and the empty vector (Mock) were also studied. After transduction we analyzed for surface TCR expression by flow cytometry to determine whether the CD3γ/δ chimeric constructs were able to reconstitute surface TCR expression (**Figures 1A, B**).

In JGN cells, γγγ, γγδ, γδδ and γγ- constructs restored TCR surface expression, whereas the empty vector (Mock), δγγ, δδδ, M1V or H29X did not. These results confirmed that the EC domain of CD3γ plays a unique role in surface TCR expression in JGN cells as previously reported (21).

In T cells from patients with CD3γ deletions that show reduced rather than absent surface TCR expression (7), the γγγ, γγδ and γδδ constructs restored TCR cell surface expression to normal levels as they did in JGN cells. Interestingly, the δγγ and γγ- constructs had opposite effect in JGN *versus* T cells from γ^—^ patients (**Figures 1A, B**). Thus, whereas the EC domain of CD3γ was indispensable for TCR cell surface expression in JGN cells, no CD3γ domain essential for restoration of normal TCR cell surface expression in T cells from patients with CD3γ deletions could be identified.

### The IC domain of CD3γ is required for PKC-mediated TCR down-regulation in both JGN cells and in T cells from γ^—^ patients

**Figure 2:**
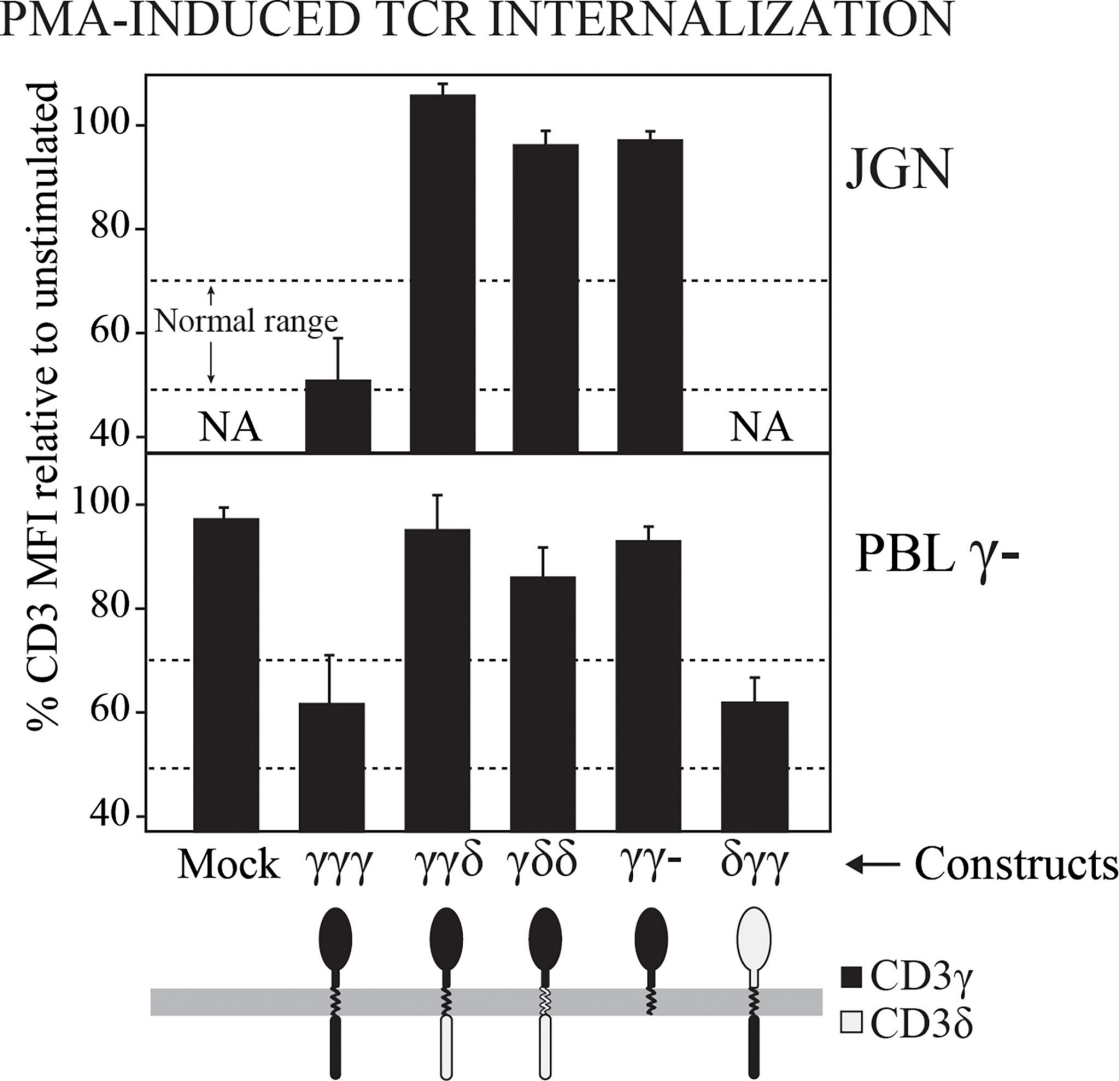
TCR internalization after PMA-mediated stimulation of PKC in γ^—^ cells (JGN or PBL) transduced with the indicated CD3 constructs. Data are expressed as % CD3 MFI relative to untreated cells. NA, Not analyzable (no surface TCR). n > 3.

It has been reported that the IC domain of CD3γ contains a motif that is involved in TCR down-regulation following PKC activation in JGN cells (31). We wanted to investigate whether this was also the case in T cells from γ^—^ patients. To this end, PMA-induced TCR internalization was studied in cells transduced with the indicated CD3 constructs. We found that all the T cells lacking the IC domain of CD3γ had impaired PKC-mediated TCR down-regulation, confirming that the IC domain of CD3γ was required for PKC-mediated TCR down-regulation in both JGN cells and in T cells from γ^—^ patients (**Figure 2**). Although unable to restore surface TCR in JGN, the δγγ construct containing the IC domain of CD3γ was fully functional for PMA-induced down-regulation in γ^—^ patient T cells.

### The IC domain of CD3δ is associated with increased numbers of nanoclustered TCRs

**Figure 3:**
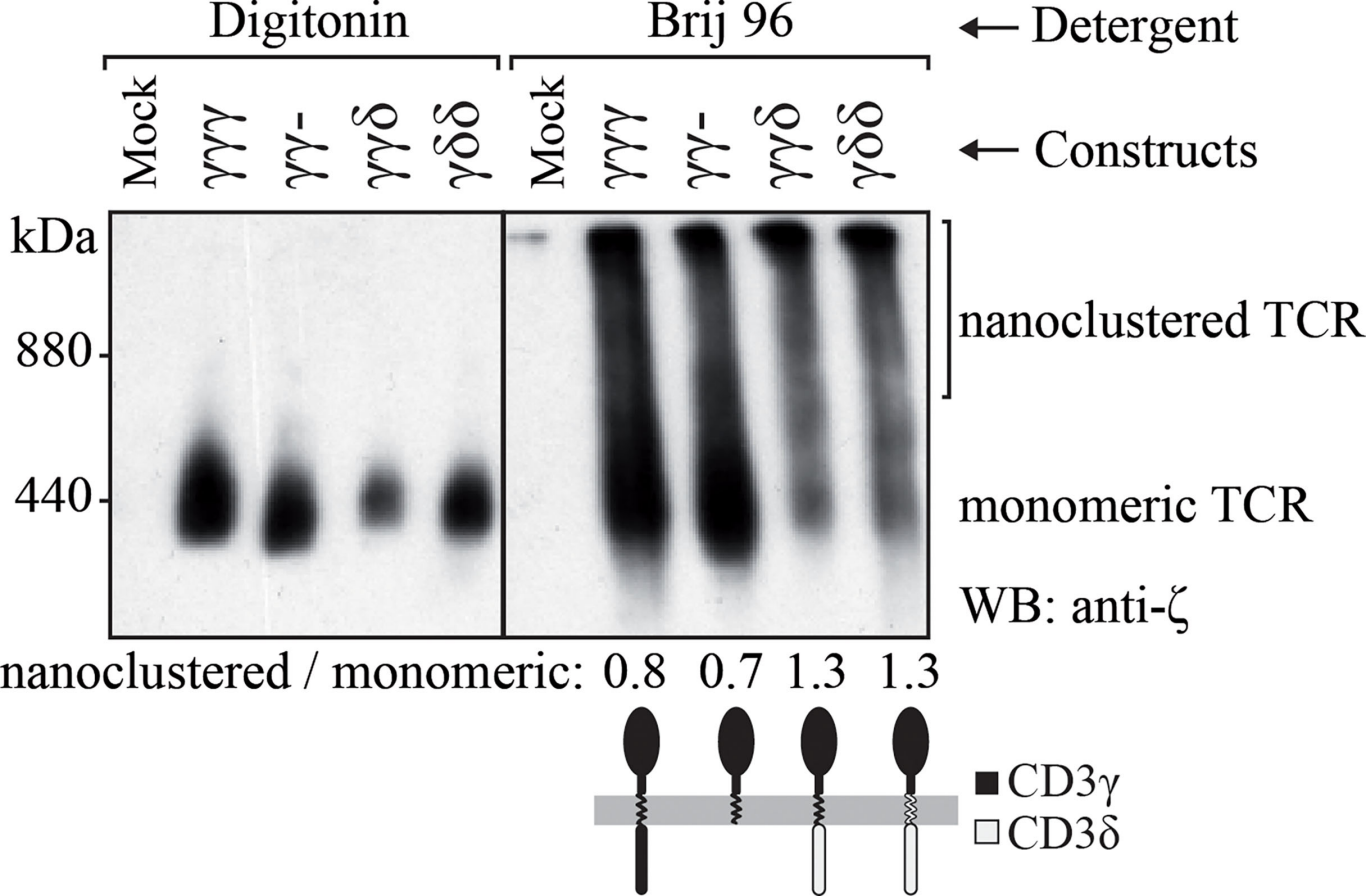
TCR nanoclustering in JGN cells transduced with the indicated constructs. TCR nanoclusters are disrupted (left) or preserved (right) by different detergents as shown after BN-PAGE and anti-ζ immunoblotting. Numbers at the bottom indicate nanoclustered/monomeric proportions by densitometry in each Brij96V lane. n=2.

**Figure 4:**
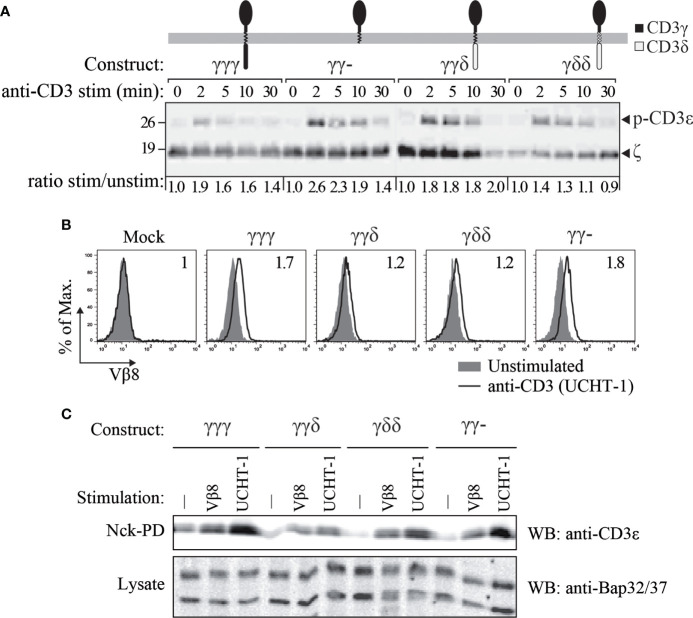
CD3ε phosphorylation **(A)** and TCR binding to Nck **(B, C)** in JGN cells transduced with the indicated constructs. **(A)** CD3ε phosphorylation was studied using rabbit anti-phospho-CD3ε-Y1 antisera at different time points after TCR engagement with 5 μg/mL anti-CD3ε mAb (UCHT-1). Numbers at the bottom indicate CD3ε phosphorylation relative to time 0 by densitometry, normalized to the loading control in each lane (ζ). **(B)** Cells were stimulated as in A (empty histograms) for 5 min at 37°C and compared with unstimulated cells (filled histograms). Cell lysates were incubated with GST-Nck^SH3.1^ fusion protein-coupled beads. After the pull down, the beads were probed with anti-TCR Vβ8 antibodies and analyzed by flow cytometry. The CD3 conformational change was revealed by a shift in MFI. Numbers in each histogram indicate stimulated/unstimulated MFI ratios. **(C)** Cells were left untreated (-) or stimulated as in A (UCHT-1) or with an anti-TCR Vβ8. After lysis, lysates were incubated with GST-Nck^SH3.1^ coupled beads to pull down TCRs that underwent the CD3 conformational change. n=2.

Surface TCR complexes are expressed as a combination of monomeric TCRs with the TCRαβ-CD3εδ-CD3εγ-ζζ stoichiometry and high molecular weight nanoclusters thereof of different sizes (28, 32, 33). It was shown that this nanoclustering is influenced by the invariant chains, as TM mutations in ζ can strongly reduce the nanoclusteres (34). The observation that increased numbers of CD3δ domains seemed to correlate with decreased TCR cell surface expression in JGN cells (**Figure 1**) prompted us to study their nanoclustering by BN-PAGE. To analyze the size distribution of the TCRs expressed in each transduced cell line, we lysed the cells with two different detergents, a mild detergent that preserves TCR oligomers (Brij96V) and a stronger detergent that disrupts these interactions (digitonin) (28, 34). As expected, in digitonin we only detected the monomeric TCRs. In contrast, in Brij96V monomeric and nanoclustered TCRs were seen in each cell line tested (**Figure 3**). In JGN cells transduced with constructs carrying the IC domain of CD3δ the ratio of nanoclustered to monomeric TCRs was clearly increased, suggesting that the CD3δ IC domain promoted nanocluster formation. This did not seem to correlate with ITAM phosphorylation, as shown for CD3ε (**Figure 4A**). Unfortunately, we could not perform these experiments in T cells from γ^—^ patients due to limited cell numbers.

### An IC domain at CD3γ is dispensable for proximal TCR signaling but required for IL-2 and TNF-α production

The CD3δ-influenced differences in JGN surface TCR expression (**Figure 1**) and nanoclustering (**Figure 3**) begged the question of how the different reconstituted TCR complexes transduced signals after engagement.

Next, tyrosine phosphorylation of CD3ε, which is a very early TCR-proximal activation event, was evaluated (**Figure 4A**). To this end, the JGN transductants were stimulated with anti-CD3 antibody and consequent phosphorylation was measured by SDS-PAGE and Western blotting using specific antibodies. We found that in all TCRs, namely the ones with γγγ, γγ-, γγδ and γδδ, CD3ε was phosphorylated after TCR stimulation.

The TCR exists in two different conformations; the resting, in active conformation and the active, open conformation (26, 35). The latter one is stabilized by antigen- or antibody-binding and allows phosphorylation of the TCR, due to the exposure of the cytoplasmic tails (4, 36). This CD3 conformational change allows a proline-rich sequence in the cytoplasmic tail of CD3ε to bind to the SH3.1 domain of the adaptor protein Nck (26); and this binding is used as a readout for this structural change. Hence, the CD3 conformational change was detected by a pulldown using GST-Nck^SH3.1^ fusion protein-coupled beads, followed by either flow cytometry (**Figure 4B**) or western blotting (**Figure 4C**). The results showed that all constructs allowed the TCR to switch to the Nck^SH3.1^-binding conformation upon anti-TCRβ or anti-CD3 antibody binding. Thus, absence of the cytoplasmic tail of CD3γ did not prevent the TCRs from undergoing the CD3 conformational change and thus allowed CD3 to be phosphorylated.

From these results, we concluded that, as long as surface expression was restored, early signal transduction through the TCR across the plasma membrane was normal in reconstituted JGN T cells, irrespectively of the composition of the CD3 chain that replaced CD3γ.

**Figure 5:**
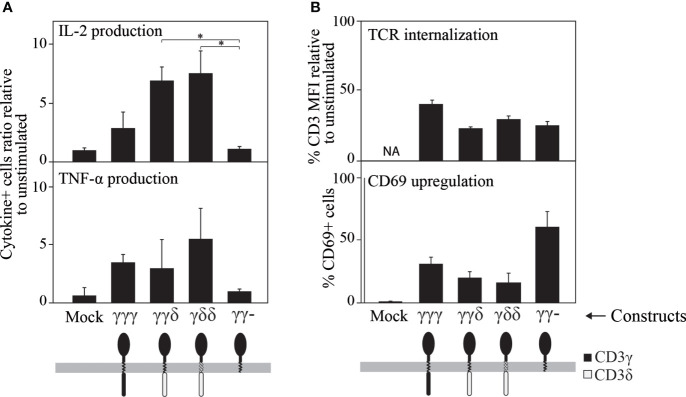
Ligand-induced cytokine secretion **(A)**, TCR internalization and CD69 expression **(B)** in JGN cells transduced with the indicated constructs (all of which restored TCR surface expression, see **Figure 1B**, top). **(A)** Cytokine secretion was studied by intracellular staining using specific antibodies 8 h after TCR engagement with 10 μg/mL anti-CD3ε mAb (UCHT-1) and represented as the ratio of % cytokine^+^ cells relative to unstimulated controls (1 means no response). **(B)** Cells were stimulated as in A using the anti-CD3 antibody Leu4, and CD3 or CD69 expression were analyzed 24 h later by flow cytometry. The results are shown as % CD3 MFI or as % CD69^+^ cells relative to unstimulated cells. n>3. Similar results were found when results were normalized to the surface TCR expression levels shown in **Figure 1B** for JGN (see **Supplementary Figure S2**). * p<0.05.

Further, late TCR-induced T-cell activation events were measured after 8 h of TCR triggering, namely cytokine production (**Figure 5A**). Quantification of IL-2 and TNF-α showed differences among the constructs, with γγ- inducing a very poor response compared to γγγ, γγδ or γδδ.

In addition, two later TCR-distal events (24 h) were measured, namely CD69 induction and ligand-induced TCR internalization (**Figure 5B**). In contrast to cytokine synthesis, CD69 expression and TCR internalization after TCR engagement were normal for all constructs including γγ-, which scored highest for CD69 induction, indicating that it was capable of some functional signal transduction. From these results, we concluded that in reconstituted JGN T-cells, the IC domain of CD3γ is required for efficient translation of proximal TCR signals into some distal T cell effector functions such as cytokine secretion, but not for other functions such as CD69 induction.

## Discussion

CD3γ deficiency, in contrast with other CD3 immunodeficiencies, allows in humans for the selection of substantial numbers of polyclonal peripheral T cells, which expressed low levels of (at least partially) functional TCR complexes. Therefore, the biological role of CD3γ within the TCR/CD3 complex is clearly different from that of the very homologous CD3δ chain (67% amino acid homology), since in CD3δ-deficient humans T cells do not develop. In this work we have addressed the biological role of the CD3γ chain in the expression and function of TCR. To this end we have analyzed the structural and functional consequences of the expression of different CD3γ/CD3δ chimeric or truncated constructions in γ^—^ patient-derived T cells or in the JGN cell line.

In JGN, which lacks CD3γ and expression of a TCR on the cell surface, we have observed that the EC domain of CD3γ, but not CD3δ, was necessary and sufficient to re-express the TCR complex. The CD3γ chain contains two N-linked glycosylation sites in the EC domain. (37). It was demonstrated that mutation of glycosylation sites in the transferrin receptor profoundly impairs its surface expression (38) but the role of CD3γ glycosylation in assembly, intracellular transport, or expression of the TCR is still unclear.

JGN cells expressing γγ− showed higher levels of the TCR on their surface, although the difference was not statistically significant in **Figure 1B**. However, it was consistently detected by different other anti-TCR/CD3 antibodies (WT31, UCHT-1, OKT3, F101.01, CRIS7, and MEM57, see **Supplementary Figure S1**). Previous studies on chimeric molecules containing the di-leucine-based endocytosis motif of CD3γ (39) have indicated that ζ can mask this motif. Successive truncations of the cytoplasmic tail of ζ led to reduced TCR surface expression levels. The reduced TCR levels were caused by an increase in the TCR endocytic rate in combination with an unaffected exocytic rate. Furthermore, TCR degradation was increased in cells with truncated ζ. Introduction of CD3γ with a disrupted di-leucine-based endocytosis motif partially restored TCR expression in cells with truncated ζ chains, indicating that ζ masks the endocytosis motif in CD3γ and thereby stabilizes TCR surface expression. Also, when appended to the human CD4 EC and TM domains, the di-leucine motif of the IC domain of CD3γ reduces the surface expression of such chimeric 44γ protein, but not of a di-leucine-less truncated version termed 44γ, in the non-T-cell line CHO (40). We believe a similar mechanism might explain the observed higher TCR expression in JGN cells transduced with γγ−.

On the other hand, we observe a disparity in terms of the γγ− construct restoring surface TCR expression in JGN cells but not in γ— PBLs. Conversely, the effect of the δγγ construct on surface TCR expression in JGN *vs* γ— PBLs is also strikingly different (**Figure 1B**). JGN cells were produced by mutagenesis and selected to show no surface TCR, under the misconception that CD3γ is strictly required for TCR expression. Indeed, reconstitution of JGN with CD3γ showed that wild-type TCR levels were not reached (**Figure 1A**, top), indicating that JGN likely has additional mutations affecting TCR regulation. In contrast, natural CD3γ-deficient T-cells were selected *in vivo* by the patient`s thymus from a diverse and polyclonal repertoire of immature T cells, with differently rearranged variable TCR chains, so that those expressing sufficient surface TCR can be selected to reach the peripheral blood (10) and thus express considerable amounts of surface TCR that reach normal levels after CD3γ transduction (**Figure 1A**, bottom). This fundamental difference may explain the disparities observed in **Figure 1B**).

Thus, CD3γ-deficient T-cells constitute a more physiological system to address some of our claims. Unfortunately, they are extremely limited, and we could not use them to address some of the biochemical and functional studies performed in JGN.

Functional analyses showed that the IC domain of CD3γ was essential for PMA-induced TCR downmodulation in both Jurkat and natural γ— reconstituted T cells and could not be replaced by the IC domain of CD3δ (21, 39).

On the other hand, the normalization of surface TCR expression requires at least the presence of the EC or IC domain of CD3γ within a complete CD3 construct since it is achieved with all constructs except IC-less CD3γ or endogenous CD3δ. This discrepancy could be due to the different origin of JGN and natural mutants. Some experiments have indicated that the EC domains of the TCR chains are involved in assembly of the TCR. One study demonstrated that the association of CD3 chains with TCRαβ was stabilized by the EC constant domain of the TCRβ chain (39). In another experiment it was shown that the assembly of TCRα and TCRβ was dependent on the EC domains of these chains (3). Both experiments involved the use of COS cells. As different conditions for TCR assembly might exist in non-T cells compared with T cells, conclusions on TCR assembly based on results obtained with non-T cells should be drawn with caution. This also applies to JGN and natural mutants.

Interestingly, JGN-γγδ expressed less TCR as compared to JGN-γγ- and likely JGN-γγγ (**Figure 1**), although it signaled quite well (**Figures 4A**, **5A**). This could be due to its higher ratio of nanoclustered to monomeric TCR (**Figure 3**). Indeed, individual TCRs in nanoclusters show cooperativity leading to increased signaling (34, 41, 42). We believe that less surface TCR leads to more nanoclustered TCR, as observed in mutant ζ transfectants (43). This is interesting, because normally you expect the opposite: the higher the density of receptor on the membrane the more aggregates. Our results would suggest that there is a limiting factor for nanoclustering, and when it is used up only monomeric forms appear.

**Figure 6:**
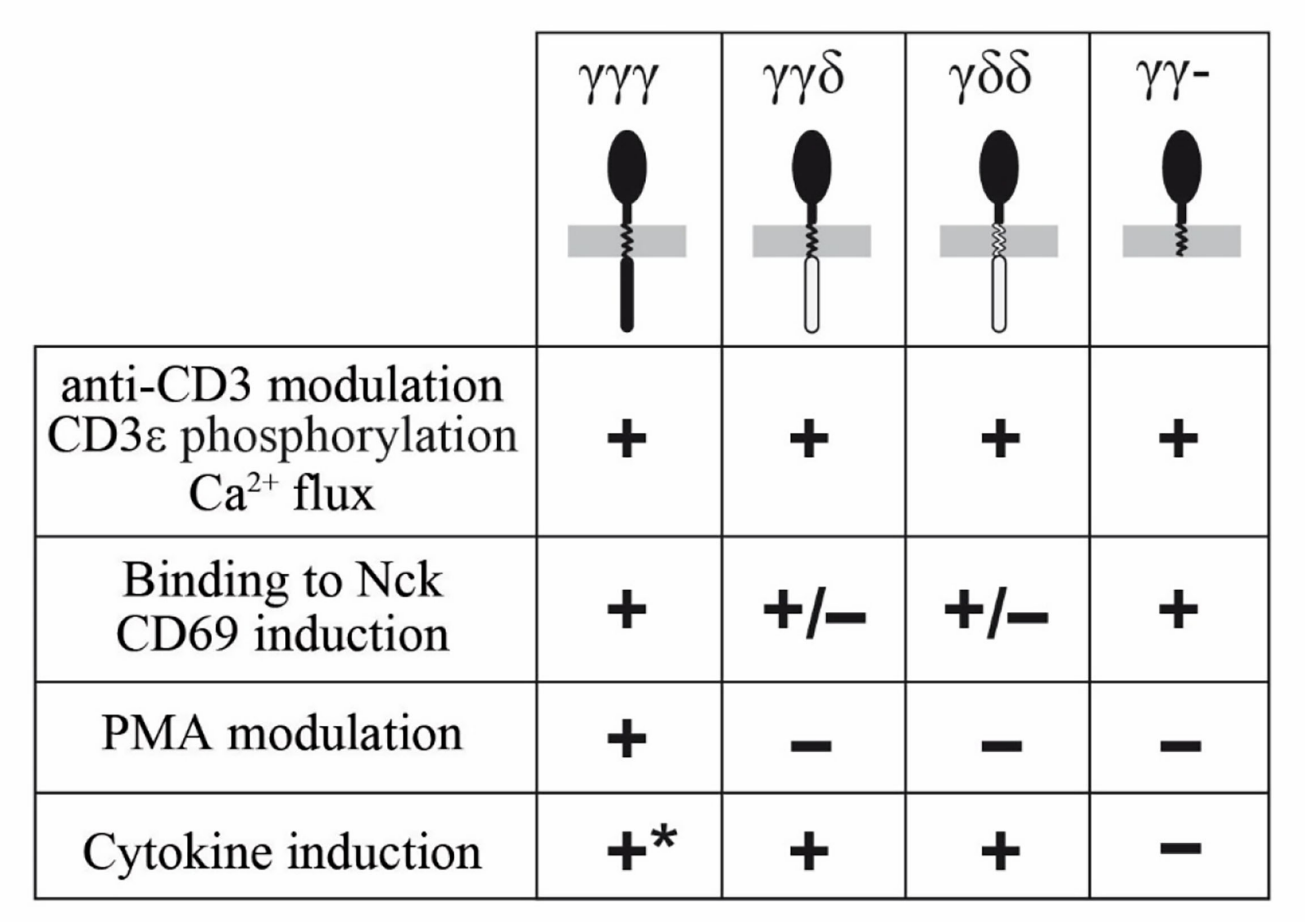
Summary of functional competence of the indicated chimeras.

Early signaling through reconstituted TCRs was normal in all tested chimeras, as measured by CD3ε phosphorylation, **Figure 6**. Further functional analyses of the chimeras showed that the TM and IC CD3δ domains could replace the TM and IC CD3γ domains, although they showed some early signalling differences such as Nck recruitment, where IC CD3δ seemed to be less effective, or CD69 induction, where IC or TM CD3δ impaired induction. In addition, we observed a contradictory result: the IC domain of CD3γ was necessary for cytokine secretion in JGN cells, as γγ− is the only construct that showed poor response in comparison to the other constructs. However, this construct produced the highest number of CD69+ cells. Since the regulation of both pathways is different, with cytokine production being more complex than CD69 induction, a possible explanation could be that the ITAM requirement may be different for the activation of those pathways. We could speculate that the intracellular domain of CD3γ has an inhibitory effect on CD69 induction. Differential induction of activation pathways by TCR lacking the CD3γ ITAM has been reported previously (44), including normal CD69 upregulation and ZAP70 activation, but severely impaired Erk or LAT phosphorylation.

The IC domains of CD3δ and CD3γ were indistinguishable for other early functional parameters, such as TCR modulation by CD3 antibodies, or late functional parameters (IL-2 and TNF-α production, **Figure 6**).

## Data availability statement

The original contributions presented in the study are included in the article/**Supplementary Material**, further inquiries can be directed to the corresponding author/s.

## Ethics statement

The studies involving human participants were reviewed and approved by CEIm Hospital Clínico San Carlos. The patients/participants provided their written informed consent to participate in this study.

## Author contributions

BG, AG, VP-F, and JR conceived the experimental study and wrote the manuscript. AG designed and performed most of the experiments and analyzed the data. VI and CJ were involved in some experiments. CG contributed to design of the cDNA clones and writing the manuscript. WS contributed to design of several experiments and to the writing of the manuscript. AVM, RFM, MH-A, and JM-F contributed with technical help and manuscript elaboration and submission. All authors participated in the manuscript revision before submission. All authors contributed to the article and approved the submitted version.

## Funding

This work was supported by grants from the Ministerio de Economía y Competitividad (MINECO PID2021-125501OB-I00 and RTI2018-095673-B-I00), Comunidad Autónoma de Madrid (CAM B2017/BMD3673) and Asociación Española Contra el Cáncer (AECC PROYE20084REGU). RFM was supported by a MINECO scholarship (FPU19/03136). WS was supported by the German Research Foundation (DFG) through BIOSS - EXC294 and CIBSS - EXC 2189 and SFB1381 (A9 to WS). VI and CJ were supported by the DFG through GSC-4 (Spemann Graduate School).

## Conflict of interest

The authors declare that the research was conducted in the absence of any commercial or financial relationships that could be construed as a potential conflict of interest.

## Publisher’s note

All claims expressed in this article are solely those of the authors and do not necessarily represent those of their affiliated organizations, or those of the publisher, the editors and the reviewers. Any product that may be evaluated in this article, or claim that may be made by its manufacturer, is not guaranteed or endorsed by the publisher.
