# Supplementary material for: The role of the different CD3γ domains in TCR expression and signaling
Source: Front Immunol. 2022 Sep 2;13:978658. doi: 10.3389/fimmu.2022.978658 (PMC9478619; doi:10.3389/fimmu.2022.978658)
Supplement: Supplementary file 1 [file DataSheet_1.docx]

Supplementary Figures

**
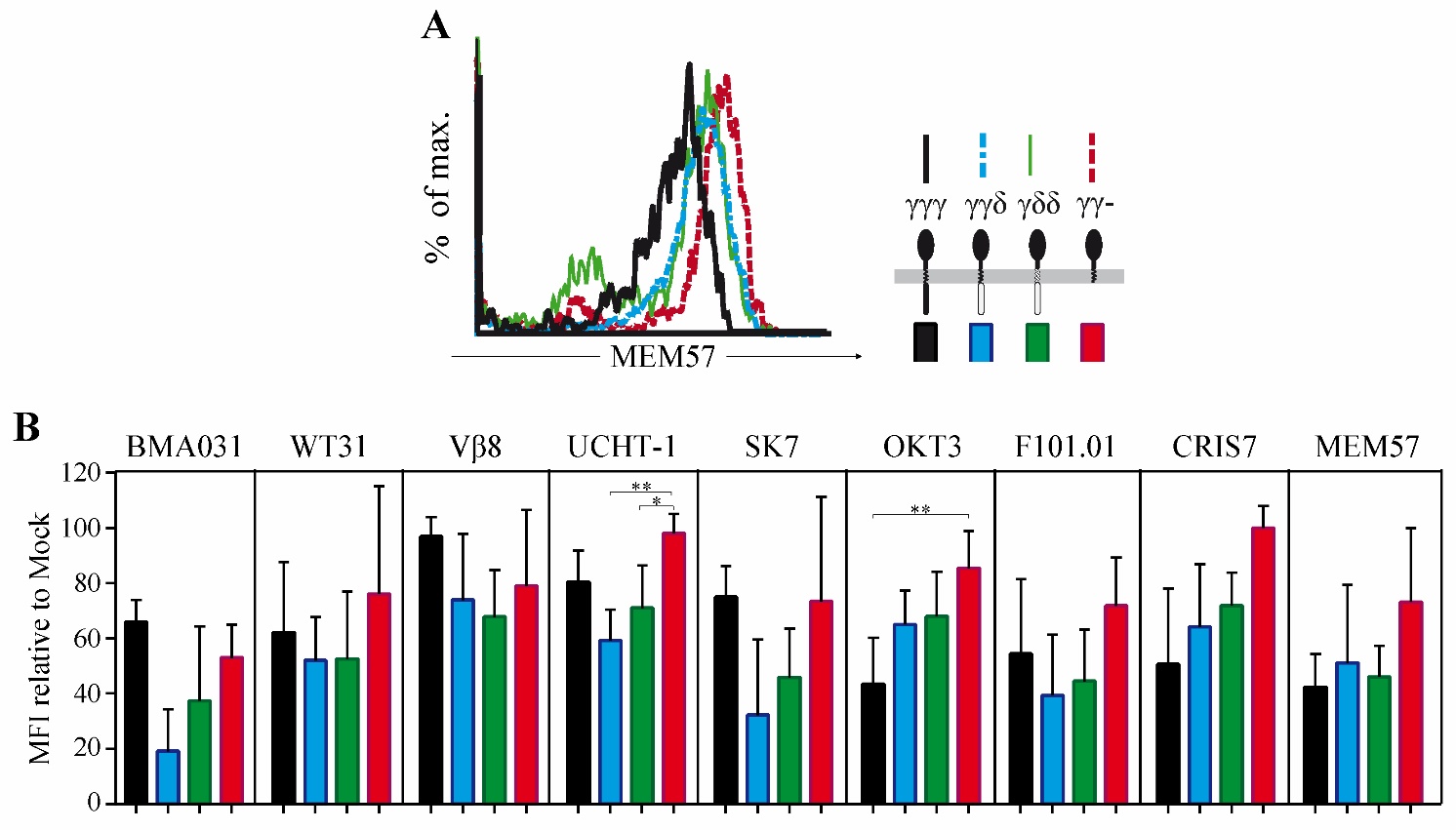
**

**Supplementary Figure S1.** Surface TCR expression determined by flow cytometry in JGN γ^—^ cells transduced with the indicated CD3γ/δ constructs. **(A)** Representative CD3 expression histograms using MEM57 antibody in cells transduced with the indicated constructs. **(B)** TCR mean fluorescence intensity (MFI) using the indicated mouse monoclonal antibody clones against TCRαβ (BMA031, WT31 and Vβ8) or CD3εδ/εγ (UCHT-1, SK7, OKT3, F101.01, CRIS7 and MEM57) relative to Mock transduced cells ± SEM in 3-6 independent experiments. * *p*<0.05, ** *p*<0.01.

**
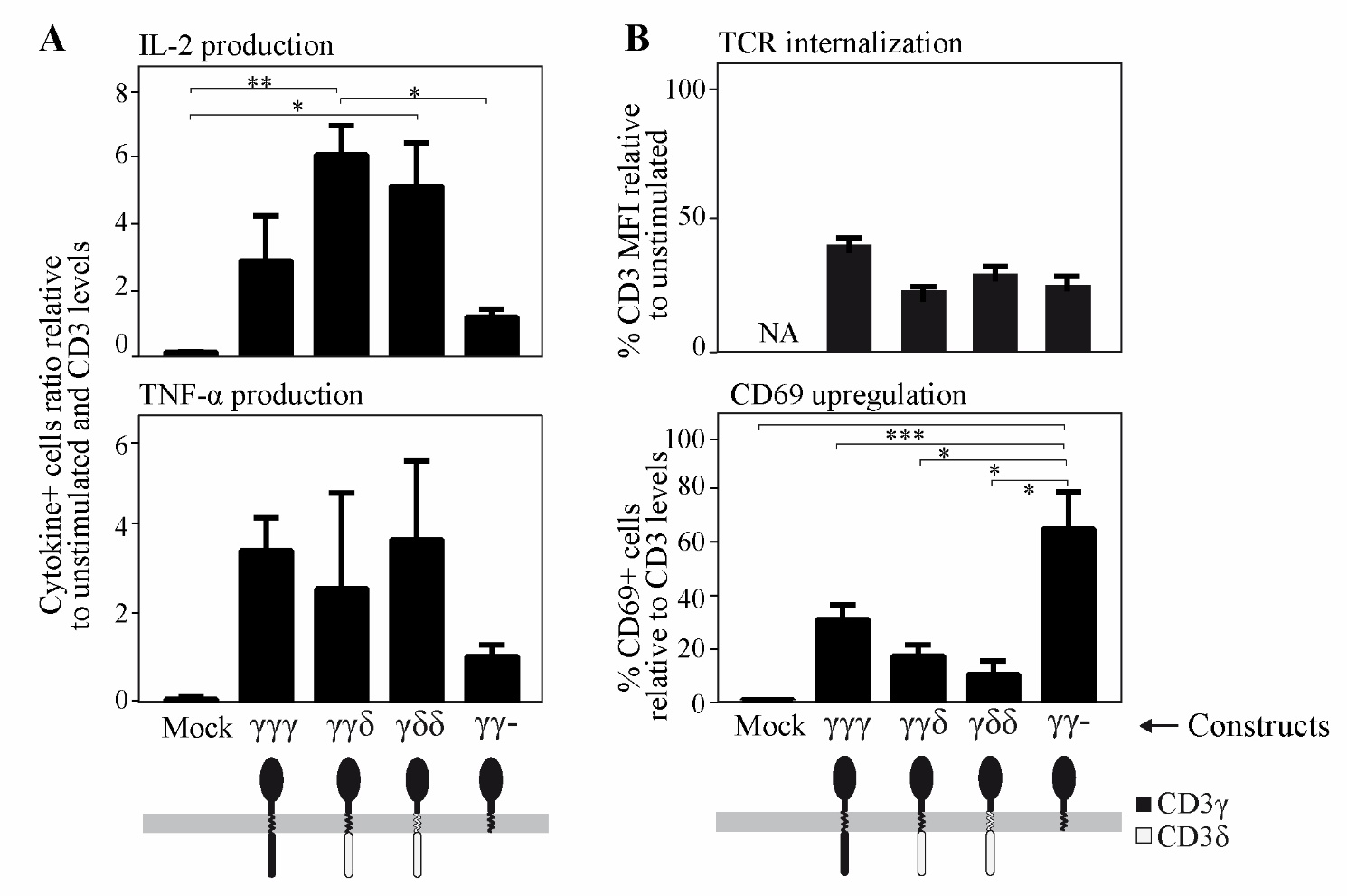
**

**Supplementary Figure 2.** Ligand-induced cytokine secretion **(A)**, TCR internalization and CD69 expression **(B)** in JGN cells transduced with the indicated constructs (all of which restored TCR surface expression, see Fig. 1B, top), normalized to the the surface TCR expression levels. (**A)** Cytokine secretion was studied by intracellular staining using specific antibodies 8 h after TCR engagement with 10 μg/mL anti-CD3ε mAb (UCHT-1) and represented as the ratio of % cytokine^+^ cells relative to unstimulated controls (1 means no response). (**B)** Cells were stimulated as in A using the anti-CD3 antibody Leu4, and CD3 or CD69 expression were analyzed 24 h later by flow cytometry. The results are shown as % CD3 MFI or as % CD69^+^ cells relative to unstimulated cells. n>3. * *p*<0.05, ** *p*<0.01, *** *p*<0.001.
